# Laser-Fabricated APTES-Functionalized Interdigitated Electrodes with a Portable Readout: A Proof-of-Concept Impedimetric Response to Aqueous Nosema Spore Suspensions

**DOI:** 10.3390/bioengineering13101120

**Published:** 2026-09-25

**Authors:** Kamil Çınar, Christopher Mayack, Hülya Eraslan-Gültekin, Ali Fuat Ergenç, Melike Kefeli, Asiye Gök Yurttaş

**Affiliations:** 1 Faculty of Science, Department of Physics, Gebze Technical University, 41400 Kocaeli, Türkiye; kcinar@gtu.edu.tr; 2 Department of Biology, College of Science and Health, William Paterson University, Wayne, NJ 07470, USA; cmayack@gmail.com; 3 Department of Control and Automation Engineering, Faculty of Electrical and Electronic Engineering, Istanbul Technical University, 34469 Istanbul, Türkiye; eraslanhu@itu.edu.tr (H.E.-G.); ali.ergenc@itu.edu.tr (A.F.E.); 4 Department of Electrical and Electronic Engineering, Erzincan Binali Yıldırım University, 24002 Erzincan, Türkiye; 5 Department of Neuroscience, Aziz Sancar Institute of Experimental Medicine, Istanbul University, 34093 Istanbul, Türkiye; melikefeli@gmail.com; 6 Department of Medical Biochemistry, Faculty of Medicine, Istanbul Atlas University, 34408 Istanbul, Türkiye

**Keywords:** impedimetric transducer, *Nosema* spp., impedance spectroscopy, honeybee health, APTES functionalization

## Abstract

Nosemosis, caused by the microsporidian parasites *Nosema apis* and *Nosema ceranae*, is a major contributor to honeybee (*Apis mellifera*) colony losses, yet its diagnosis still relies on destructive sampling followed by light microscopy or nucleic-acid amplification in a laboratory. We describe an impedimetric transducer intended as a first step toward a non-destructive alternative. Interdigitated gold electrodes were patterned on standard microscope slides by nanosecond laser ablation, a route that requires neither photolithography nor cleanroom processing, and were functionalized with (3-aminopropyl)triethoxysilane (APTES) deposited from either ethanol or toluene. Because the 0.01–50 MHz measurement window lies far above the range in which faradaic charge transfer contributes measurably, the response is non-faradaic and is governed by the permittivity and the ionic conductivity of the deposited droplet rather than by interfacial charge transfer. Films deposited from ethanol gave the largest and most reproducible contrast between an aqueous spore suspension (1500 spores/µL) and a deionized-water control. At the structural resonance of the electrode (≈16 MHz) the contrast in impedance magnitude is approximately 10% and is not resolved above the chip-to-chip scatter, which is expected because the magnitude at resonance is set by the series resistance of the electrode path rather than by the droplet. The two liquids are instead separated by the reactive component: the imaginary part of the impedance differs by 15.7 ± 1.8 MΩ near 6.8 MHz, about nine times the replicate uncertainty, and the phase by 33 ± 7° at 4.5 MHz. A self-contained readout built around an ESP32 microcontroller reproduced the direction of this change, its output voltage rising by approximately 33% after the suspension was applied. The scope of these observations is deliberately narrow: a single spore concentration in deionized water, no spore-free filtrate control, no calibration series, no selectivity panel and no measurement in a fecal matrix. The contribution is accordingly methodological—a cleanroom-free fabrication route, a quantified solvent dependence of the APTES film, and the transfer of the measurement from a benchtop spectrometer to a portable unit—rather than a validated diagnostic assay.

## 1. Introduction

Sensors lie at the core of modern measurement systems, enabling the detection of environmental, biological, and physical phenomena across a wide range of applications [1]. Rapid advancements in sensor technology have catalyzed innovations in environmental monitoring, medical diagnostics, industrial automation, aerospace, and defense systems [2]. Among critical ecological contributors, honeybees (*Apis mellifera*) serve as keystone generalist pollinators, providing invaluable services to natural ecosystems and global agriculture [3,4,5,6]. However, honeybee colonies are increasingly threatened by a range of abiotic and biotic stressors, among which infections caused by *Nosema* species are among the most detrimental [7,8,9,10]. *Nosema apis* and *Nosema ceranae* are obligate intracellular microsporidian parasites that infect the midgut of honeybees and are known to contribute to colony collapse and productivity loss [11,12]. As members of the phylum Microsporidia, *Nosema* spp. are notoriously difficult to detect owing to their microscopic size and intracellular nature [13].

Traditional diagnostic techniques for *Nosema* infection are labor-intensive, invasive, unreliable, or require specialized laboratory facilities. Beekeepers often rely on crude and unreliable methods such as visual assessment of dissected bee guts or of fecal characteristics. Even professional diagnostics based on microscopic examination or molecular analysis involve complex sampling, frequently requiring the sacrifice of bees, DNA extraction, and long processing times [14,15,16,17,18]. These limitations underline the urgent need for a non-invasive, rapid, reliable, and cost-effective biosensing method for the real-time detection of *Nosema* spores under field conditions.

Electrochemical impedance-based biosensing has been successfully applied to the detection of bacterial pathogens, including *Escherichia coli*, using functionalized electrode interfaces [19]. Among such impedimetric approaches, transducers built on interdigitated electrode (IDE) arrays have become a widely used route to label-free, low-cost sensing, because the coplanar finger geometry confines the electric field to a thin layer immediately above the substrate and therefore couples strongly to any change in the permittivity or the conductivity of the adjacent liquid. The geometry has been exploited both in faradaic configurations, in which a redox couple reports on charge-transfer resistance [20], and in redox-free configurations, in which the measured quantity is the interfacial capacitance of a functionalized surface [21,22]. In parallel, direct laser patterning has emerged as a cleanroom-free alternative to photolithography for defining metal electrodes, with gold structures written onto glass or onto laminated gold leaf in seconds and at feature sizes of tens of micrometres [23]. Diagnosis of nosemosis, by contrast, still rests almost entirely on destructive laboratory assays—haemocytometer counting, multiplex and quantitative PCR, digital PCR and isothermal amplification—each of which requires the bees to be sacrificed and the sample to be processed off-site [24,25]. To our knowledge no impedimetric platform for *Nosema* has been reported. The novelty of the present work therefore lies in three elements taken together: a photolithography-free laser route for producing interdigitated gold electrodes on ordinary microscope slides; a controlled comparison of two APTES deposition solvents on nominally identical transducers, supported by contact-angle and electron-microscopy data; and a self-contained microcontroller readout that resolves the resulting impedance contrast without laboratory instrumentation. The importance of establishing this combination is practical: any future field assay for nosemosis will have to be built on a transducer that can be made cheaply and interrogated with simple electronics, and the present study asks whether such a transducer is physically capable of registering the analyte at all.

As a first step toward addressing this gap, we present an impedimetric sensing platform and evaluate its response to aqueous Nosema spore suspensions. This microfabricated transducer employs impedance spectroscopy on interdigitated electrode (IDE) arrays functionalized with an organosilane, specifically (3-aminopropyl) triethoxysilane (APTES), in order to enhance binding interactions with fungal spores [26]. APTES has previously been employed for spore immobilization owing to its ability to self-assemble into amine-reactive films on glass and other inorganic substrates [27]. Such surface modification enhances sensor repeatability, sensitivity, and overall performance. The motivation for the present work rests on a straightforward physical premise: because *Nosema* spores possess dielectric and ionic characteristics that differ from those of the carrier liquid, their presence at an interdigitated electrode interface should modify the interfacial impedance in a manner that impedance spectroscopy can resolve. Provided that this modification is large enough and sufficiently reproducible, it can serve as a direct, decisive, label-free indicator of infection during field use, and can be captured by simple, low-cost electronics rather than by a laboratory spectrometer. It is important to state at the outset what the present study does and does not establish. APTES provides a general amine-terminated surface rather than a molecular recognition element directed against Nosema, so the platform described here is an impedimetric transducer rather than a biosensor in the strict sense of the term, and we use the former designation throughout. All measurements reported below were made on spore suspensions in deionized water at a single spore concentration; no measurement in fecal matrix, no concentration series and no non-target control are presented. Accordingly, the contribution of this work lies in three areas: a laser-based fabrication route for interdigitated gold electrodes that requires no photolithography; a systematic comparison of APTES films deposited from ethanol and from toluene, supported by contact-angle and scanning electron microscopy data; and the demonstration that the resulting impedance contrast is large enough to be resolved by a self-contained microcontroller-based readout rather than a laboratory spectrometer. Whether that contrast is specific to Nosema spores is a question that the present dataset cannot answer, and we return to it explicitly in Section 3.

## 2. Materials and Methods

### 2.1. Materials, Instrumentation and Biological Material

**Reagents**: (3-Aminopropyl)triethoxysilane (APTES, 99%), anhydrous toluene (99.8%), absolute ethanol, acetone, and isopropyl alcohol were obtained from Merck (Darmstadt, Germany) and used as received. Deionized water (resistivity 18.2 MΩ·cm at 25 °C) was produced using a Milli-Q^®^ water purification system (Merck Millipore, Burlington, MA, USA). Standard soda-lime microscope slides (1.1 mm thickness) were supplied by ISOLAB Laborgeräte GmbH (Eschau, Germany). Gold (99.99% purity) was obtained from Kurt J. Lesker Company (Jefferson Hills, PA, USA), and high-purity nitrogen (99.999%) was supplied by Linde Gas (Pullach, Germany). Phosphate-buffered saline (PBS) used for spore handling was obtained from Merck (Darmstadt, Germany).

**Instrumentation:** Gold films were deposited with a Univex 450 thermal evaporator (Leybold GmbH, Köln, Germany). Electrode patterning was performed with a 20 W MOPA nanosecond fibre laser marker (FiberLAST A.Ş., Ankara, Türkiye). Oxygen plasma treatment was performed using an oxygen plasma surface activation, cleaning, and descumming system at the İTÜnano Cleanroom Facility, Istanbul Technical University (İTÜ), Istanbul, Türkiye. Surface morphology was examined with a Philips-FEI XL30 ESEM-FEG scanning electron microscope (FEI Company, Hillsboro, OR, USA) operated at 30 kV. Static water contact angles were measured using a contact-angle goniometer at the National Membrane Technologies Research Center (MEM-TEK), Istanbul Technical University, Istanbul, Türkiye. Spore counts were performed under an Olympus CH20 compound light microscope (Olympus Corporation, Tokyo, Japan). Impedance spectra were acquired with a Zurich Instruments HF2IS impedance analyzer equipped with an HF2TA transimpedance current amplifier (Zurich Instruments AG, Zurich, Switzerland). The portable unit was built around an ESP32 microcontroller (Espressif Systems, Shanghai, China), an AD9833 direct digital synthesis signal generator (Analog Devices, Wilmington, MA, USA) and an SSD1306 OLED display.

**Biological material:** Nosema spores were propagated in caged Apis mellifera workers following the procedure described previously by our group [28]. In brief, one capped brood frame was taken from each of three source colonies and held in an incubator at 32 °C and 60% relative humidity until the adult bees emerged. Newly emerged bees were removed from the frames within 24 h of emergence, pooled, and confined in three cages of forty bees each with 50% sucrose solution provided ad libitum. Each bee was inoculated with 10^5^ spores. Ten days post-inoculation the bees were pooled, homogenized, and the spores purified according to the standard methods for Nosema research [29].

The species composition of the resulting spore stock was determined by multiplex PCR on a subsample, using the primer sets of Martín-Hernández et al. [11] listed in Table 1. Each 25 µL reaction contained 4 ng of template DNA, 12.5 µL of 2× Taq PCR MasterMix (abm, Richmond, BC, Canada) and 400 nM of each primer. Cycling comprised an initial step of 95 °C for 2 min followed by 35 cycles of 95 °C for 45 s, 59.3 °C for 45 s and 72 °C for 1 min. Products were resolved at 100 V for 30 min on a 1% agarose gel stained with GelRed Nucleic Acid Stain (10,000× in water; Biotium, Fremont, CA, USA) and visualized on a Bio-Rad Gel Doc EZ station (Bio-Rad, Hercules, CA, USA). Nosema apis and *N. ceranae* positive controls and a water negative control were included in every run. Both the 218 bp *N. ceranae* fragment and the 321 bp *N. apis* fragment were detected, establishing that the stock was a mixed preparation containing both species rather than a single-species isolate.

Spore concentration was determined with a haemocytometer under light microscopy following Ptaszyńska et al. [30]. Counts were performed in triplicate on independent aliquots of the same stock and the mean value was used, giving 1.5 × 10^6^ spores/mL. The route from the pooled homogenate to this final suspension was as follows. The homogenate was clarified and the spores were sedimented by repeated centrifugation according to Fries et al. [29]; the pellet was washed in phosphate-buffered saline (PBS, 10 mM, pH 7.4) to remove gut debris and soluble homogenate, and was then washed three further times in deionized water (18.2 MΩ·cm) in order to displace the buffer before final resuspension in deionized water. The purpose of the final aqueous washes was to reduce the electrolyte carried over from purification; they cannot be assumed to remove it entirely, and the conductivity of the resulting suspension was not measured. Because the manuscript identifies residual ionic content as a possible origin of the measured signal, this handling history is stated explicitly here and its consequences are examined in Section 3. The stock was stored at 4 °C and used within 24 h of preparation. For impedance measurements, 2 µL of this stock was applied to the sensor, corresponding to approximately 3000 spores, or 1500 spores/µL.

Because the stock contained both *N. apis* and *N. ceranae*, any impedance response recorded here reflects the combined dielectric and ionic contribution of a mixed-species preparation. The two species differ in spore dimensions and surface characteristics, and the present data therefore cannot be attributed to either species individually. This is stated as a limitation in Section 3.

### 2.2. Fabrication of the Sensor Probes and Experimental Setup

Sensor electrodes were fabricated on standard 1.1 mm thick microscope glass slides that had been coated with a 100 nm gold film by thermal evaporation prior to laser structuring. Laser fabrication techniques were employed to pattern the electrodes. Using a computer-controlled laser marker, the desired patterns were created by selective ablation of the gold layer. The laser had a wavelength of 1064 nm and a pulse duration of 200 ns, and the patterns were formed by a material-removal process (Figure 1). The ablated regions left exposed glass surfaces, whereas the non-ablated regions retained the gold coating. During laser scanning, an energy fluence of 1.25 J/cm^2^ was applied. The scan speed was 1250 mm/s with a repetition rate of 200 kHz, and the focal diameter of each pulse was approximately 30 µm. The sensor layout comprised three active sensing regions, each consisting of comb-type interdigitated structures with finger widths of 200 µm and inter-finger gaps of 37 µm. These active areas measured 3 × 3 mm and were fabricated adjacently on the same substrate. During impedance measurements, only these active areas were exposed to the test solutions, ensuring consistent and localized analysis. The sensors were tested both with and without surface coatings.

To enhance binding interactions between the sensor and *Nosema* spores, the sensors were functionalized with APTES. Two different solvents—ethanol and toluene—were used for the APTES coating. A third control group of uncoated sensors (referred to as “No Coating”) was included in order to evaluate the baseline response. These control sensors underwent laser ablation and the standard cleaning procedure but were not functionalized with APTES. APTES solutions were prepared by mixing 1% (*v*/*v*) APTES in either 50 mL of toluene or 50 mL of ethanol. The coating method followed protocols reported in the literature for optimal spore immobilization and surface reactivity [27,31].

Three groups of sensors were prepared: Group 1, No Coating (laser structuring only, no chemical functionalization); Group 2, APTES + toluene; and Group 3, APTES + ethanol. Each sensor was cleaned sequentially with acetone, ethanol, and isopropyl alcohol (5 min each), followed by nitrogen drying. The slides were then exposed to oxygen plasma at 100 W for 5 min in order to hydroxylate the surface. Immediately after plasma treatment, the sensors were immersed in the respective APTES solutions for 5 min and subsequently incubated at 50 °C for 1 h. After thermal curing, (i) the toluene-coated sensors were washed four times with toluene, dried under nitrogen, then washed four times with 100% ethanol and dried again, and (ii) the ethanol-coated sensors were washed four times with 100% ethanol and dried under nitrogen (Figure 2). All sensor sets were subjected to a second thermal treatment at 60 °C for 1 h on a hotplate while stored in glass Petri dishes.

The wettability of the sensor surfaces was evaluated by static water contact angle (WCA) measurements, as presented in Figure 3. Each value is the mean of three droplets placed at independent positions on the same surface, so that the angles quoted below are means of triplicate determinations rather than single readings. The scatter across those three measurements is of the order of a few degrees. Because that spread is not negligible relative to the differences between the coatings, the values are used here only to rank the three surfaces, and no solid surface free energy, and no decomposition into polar and dispersive components, is computed from them at any point in this manuscript. The uncoated IDE sensor exhibited a WCA of 59° (Figure 3a), indicating an inherently hydrophilic surface character, which is consistent with the polar functional groups typically present on bare electrode substrates such as oxidized metal or dielectric surfaces. Upon functionalization with the toluene-based APTES solution, the WCA decreased marginally to 55° (Figure 3b), suggesting that this coating introduces only a minimal modification of the surface energy. Given that toluene is a non-polar aromatic solvent, the negligible change in wettability implies that any residual toluene-derived layer does not significantly alter the polar character of the underlying substrate, or alternatively that the coating is sufficiently thin to allow the substrate surface chemistry to dominate.

In contrast, the ethanol-coated sensor displayed a markedly lower WCA of 40° (Figure 3c), a reduction of approximately 19° relative to the uncoated reference. We report this as an empirical trend and refrain from assigning it to a particular surface group. A static contact angle reports the balance of interfacial energies of the whole surface; it does not identify which termini are present, and the present dataset contains no spectroscopic characterization of the deposited films, such as X-ray photoelectron or infrared spectroscopy, from which a silanol or amine surface density could be inferred. What may be stated is that ethanol and toluene are known to produce APTES layers that differ in thickness and in degree of siloxane condensation [32], and that the film deposited here from ethanol is the more wettable of the two. The measurable consequence for the present experiment is geometric rather than chemical, and is quantified below.

The observed trend in WCA values ethanol-coated (40°) below toluene-coated (55°) ≈ uncoated (59°) parallels the impedance data presented in the subsequent sections. It is important to be explicit about what this parallel can and cannot support. The spectra reported here are non-faradaic and were acquired between 0.1 and 50 MHz, a window in which neither charge-transfer resistance nor double-layer capacitance is accessible; the contact-angle trend is therefore not interpreted in terms of those quantities anywhere in this manuscript. The consequence that the present data do support is geometric: for a fixed droplet volume, the more wettable surface spreads the liquid over a larger fraction of the interdigitated area and so places a greater length of electrode in contact with the sample. That effect alone would lower the measured impedance, independently of any interaction between the APTES layer and the spores. The wettability measurements are accordingly presented as a characterization of the two coatings and as the identification of a confounding variable, not as mechanistic evidence for the impedimetric contrast reported in Figures 5 and 6.

One further qualification applies to the surface characterization as a whole. Neither contact-angle goniometry nor scanning electron microscopy demonstrates the presence of an APTES layer directly, because neither technique is chemically specific: the first reports the balance of interfacial energies at the surface and the second reports its topography. Direct confirmation would require a chemically specific method, most obviously X-ray photoelectron spectroscopy, from which the N 1s signal of the aminopropyl terminus and the Si 2p signal of the siloxane anchor could be quantified and an approximate coverage obtained, or infrared spectroscopy of the amine and siloxane bands. Neither was available to us within the funding and the instrument access of the present project, and neither could be arranged within the period allowed for this revision. X-ray photoelectron spectroscopy of the three surfaces, with quantification of the N 1s and Si 2p signals against an uncoated control, is the first item of the characterization programme planned for the continuation of this work. What the two techniques used here do establish is that the ethanol and the toluene treatments leave surfaces of measurably different wettability and of comparable morphology. That is sufficient to support the solvent comparison reported below, in which the two treatments are contrasted with each other and with an untreated control, but it is not sufficient to certify either the chemical identity or the surface coverage of the deposited film. The point is recorded again among the limitations in Section 3.

The prepared sensor sets were tested with *Nosema* spores under laboratory conditions. Impedance changes at the sensor resonance frequency were recorded using a dedicated electronic setup (Figure 4). Measurements were acquired with a computer-based data acquisition system and evaluated using standard descriptive statistics. During measurements, 2 µL of test solution was applied directly to the 3 × 3 mm active area of the sensor. The following three conditions were tested: (i) a baseline measurement with no solution applied (“Reference”); (ii) 2 µL of distilled water (“DI2ul”); and (iii) 2 µL of distilled water containing approximately 3000 spores, corresponding to 1500 spores/µL, prepared from a 1.5 × 10^6^ spores/mL *Nosema* stock suspension (“Nos2ul”). Combining the three sensor types with the three sample conditions yielded nine distinct measurement configurations. Each configuration was recorded three times, giving 27 individual measurements in total. The three recordings of each configuration were obtained from three separate sensor chips of the same coating type, all fabricated within a single batch; the repeats are therefore device-level replicates that characterize sensor-to-sensor reproducibility within one fabrication batch rather than the repeatability of a single device. **Statistical analysis:** Impedance magnitude at each frequency is reported as the mean of the three sensor chips measured under each condition, with the standard deviation across those chips plotted as the error bars in Figure 5. For clarity these error bars are not reproduced in the Nyquist representations of Figure 6, in which each trace is the mean of the same three chips; the chip-to-chip scatter for any given condition should therefore be read from Figure 5. Because each chip was measured under all three sample conditions, the Reference, DI2ul and Nos2ul data constitute paired observations on the same set of devices. Given that *n* = 3, differences between the deionized-water and spore-containing conditions are reported as effect magnitudes relative to the observed sensor-to-sensor scatter and are interpreted descriptively; no inferential hypothesis testing was performed.

Points at which the mean lock-in amplitude fell below the detection threshold of the acquisition chain were treated as noise-limited and excluded from the averages and from the figures. This criterion removed approximately 26% of the acquired points in the unloaded Reference condition, all of them at the low-frequency end of the window, and fewer than 0.1% of the points in the DI2ul and Nos2ul conditions.

**Cleaning between successive loadings:** Because each chip was measured under all three sample conditions, and because the portable experiment described in Section 2.4 likewise involved two successive liquid exposures of a single chip, the active area was cleaned between loadings by rinsing with deionized water, rinsing with absolute ethanol and drying under a stream of nitrogen. The following measurement was begun only once the unloaded impedance spectrum had returned to the reference trace recorded before the first loading. This procedure was applied consistently, but it was not itself validated: carry-over of adsorbed material, and any degradation of the APTES film across repeated cleaning cycles, were not quantified. The paired comparisons reported below should be read with that qualification, and it is listed again among the limitations in Section 3.

**Rationale for the measurement configuration:** The measurements reported here are non-faradaic. No redox mediator was added, and no supporting electrolyte was used, because the frequency window employed (0.01–50 MHz) lies far above the range in which faradaic charge transfer contributes measurably to the impedance. In this regime the response of an interdigitated electrode is governed by the permittivity and the bulk ionic conductivity of the material occupying the inter-electrode gap, together with the parasitic capacitance and inductance of the electrode geometry itself. A ferri/ferrocyanide couple, which is diagnostic of charge-transfer resistance at millihertz to kilohertz frequencies, would contribute no resolvable feature at megahertz frequencies and was therefore not employed. For the same reason we describe the technique as impedance spectroscopy rather than electrochemical impedance spectroscopy, and we do not report charge-transfer resistance or double-layer capacitance, since neither quantity is accessible from these data. For the same reason the Randles-type equivalent circuit conventionally fitted to faradaic impedance data is not applicable here: it represents charge transfer across an electrode-electrolyte interface, a process that makes no measurable contribution in the window employed, and the lumped R-L-C description of Section 2.3 is used instead. Deionized water rather than a buffered electrolyte was used as the carrier liquid in order to maximize the conductivity contrast produced by the added suspension; the consequences of this choice are examined in Section 3.

To prevent external interference, all measurements were conducted inside a Faraday cage at a constant room temperature of 21 °C. A 100 mV AC signal was applied to the sensor electrodes, and impedance spectra were collected over a frequency range of 0.01–50 MHz. The output signal was pre-amplified and analyzed using a Zurich Instruments HF2 impedance analyzer, and the data were transferred to a computer for analysis. The HF2TA transimpedance current amplifier was operated at a gain of 1 MV/A, corresponding to a transimpedance resistance of RTIA = 1 MΩ. Complex impedance values were derived from the lock-in amplifier quadrature voltage outputs (X and Y, in volts) according to Z = −(Vexc × RTIA)/(X + jY), where Vexc = 100 mV is the excitation voltage and j is the imaginary unit. All reported impedance magnitudes are expressed in MΩ. The scale follows from the instrument settings: with Vexc = 100 mV and RTIA = 1 MΩ, a lock-in quadrature amplitude of a few millivolts corresponds to a sensor impedance of tens of MΩ. The unit was given incorrectly as kΩ in the previous version and has been corrected throughout. All measurements for each sensor were completed within approximately 1 min.

### 2.3. Measurement of Impedance Variations and Sensor Performance

Impedance data were collected in triplicate for each of the nine measurement configurations defined by the three sensor types and the three measurement conditions described above: Reference (no sample), DI2ul (2 µL distilled water), and Nos2ul (2 µL distilled water containing ~3000 *Nosema* spores). The average impedance values obtained from the three sensor chips measured under each condition were calculated and plotted as Bode plots (impedance magnitude versus frequency, Figure 5) and as Nyquist plots (negative imaginary versus real impedance, Figure 6).

As shown in Figure 5, all sensors reached their minimum impedance magnitude |Z| at approximately 16 MHz, and this frequency region was therefore adopted as a common reference point at which the three coating chemistries could be compared on an equal footing. Regardless of coating type, all sensors exhibited their highest impedance in the unloaded reference state, with |Z| values of approximately 90 MΩ at the resonance frequency. The application of either distilled water (DI2ul) or the *Nosema*-containing solution (Nos2ul) produced a pronounced decrease in impedance magnitude, to approximately 15–20 MΩ at the same frequency, reflecting the substantially higher permittivity and ionic conductivity of the liquid-loaded interface relative to air.

The assignment of this minimum to a structural resonance of the transducer, rather than to a property of the sample, was asserted in the previous version without argument, and we set out the grounds for it here. Three features of the present data support it. First, the minimum falls at the same frequency, within the resolution of the sweep, for uncoated, toluene-APTES and ethanol-APTES chips; a feature originating in the interface chemistry would be expected to move with the coating. Second, it falls at the same frequency in the unloaded reference state and under both liquid loads, whereas a feature governed by the permittivity or the conductivity of the droplet would shift when a droplet is applied. Third, the Nyquist locus crosses the real axis in the same frequency region and continues into the lower half-plane (Figure 6b–d), which is the signature of a reversal of the sign of the reactance from capacitive to inductive and therefore of a series resonance; a dielectric or electrochemical relaxation would instead produce an arc that remains in the upper half-plane. An order-of-magnitude check points the same way: for a series resonance at f_0_ = 1/(2π√(LC)), a combined gap, substrate and fixture capacitance of the order of 100 pF together with a series inductance of the order of 1 µH contributed by the electrode fingers, the contact pads and the connecting leads places f_0_ in the neighbourhood of 16 MHz. We offer this as a consistency argument and not as a measurement. A direct validation is straightforward: repeat the sweep with a deliberately altered lead length, which should displace the minimum if the inductance is dominated by the leads, and measure the empty fixture with no chip inserted; this is included among the further work set out in Section 3.

The most distinct and consistent impedance shift between the two liquid samples was observed for the sensors coated with ethanol-based APTES (Figure 5c). These sensors exhibited a clear difference in response between distilled water and the *Nosema*-containing sample across the entire frequency range examined. In contrast, the toluene-based APTES sensors (Figure 5b) and the uncoated sensors (Figure 5a) showed less consistent differentiation between the two sample types. At the lower end of the frequency range, the *Nosema*-containing samples produced a lower impedance than distilled water alone, whereas at higher frequencies, particularly between 15 and 25 MHz, the responses to *Nosema* and to pure water became nearly indistinguishable for the toluene-APTES and uncoated sensors. Two qualifications attach to this description. First, the traces in Figure 5a cross one another over the sweep, so that the relative ordering of the Reference, DI2ul and Nos2ul curves on the uncoated chips is not preserved across the whole frequency range; the general statement that the suspension lowers the impedance holds in and below the resonance region but not at every frequency, and it is qualified accordingly wherever it appears. Second, because only a single spore concentration was measured, what these data establish for the ethanol-deposited films is a larger and more consistent contrast between the two liquids, not a greater analytical sensitivity. Sensitivity in the analytical sense denotes the slope of a calibration function, which the present dataset does not provide, and the term is therefore not applied to these results.

One important observation is that the test droplets spread more uniformly across the ethanol-coated sensors, providing a more homogeneous interaction with the active sensing area. This behavior is consistent with the greater hydrophilicity of the ethanol-deposited APTES films documented by the contact-angle measurements above (40° versus 55° and 59°) and with previous reports [26]. It also identifies a confounding factor that must be stated explicitly. A more hydrophilic surface causes a fixed 2 µL droplet to spread over a larger area of the interdigitated structure, thereby increasing the electrode area in contact with the liquid. Part or all of the superior contrast obtained with the ethanol-deposited films may therefore reflect this geometric effect rather than an improved chemical interaction between the APTES layer and the spores. Distinguishing between the two requires normalization of the response to the wetted area, for example by imaging the droplet footprint on each coating and expressing impedance per unit wetted length of electrode, or by confining the droplet to a fixed area with a hydrophobic ring. Neither was done here, and the solvent comparison should be read accordingly. The magnitude contrast between the two liquids at the resonance frequency is defined as 100 × (|Z|DI − |Z|Nos)/|Z|DI and is evaluated from the averaged spectra of Figure 5. On the ethanol-coated sensors it is approximately 10% at the 16 MHz reference point. Propagating the chip-to-chip standard deviations of Figure 5 through that expression gives an uncertainty of approximately 13% on the contrast itself, so the magnitude contrast at resonance is not resolved above the replicate scatter; the corresponding values are approximately 0% on the toluene-coated and approximately −9% on the uncoated sensors, neither of which is resolved either. The figure of 15% quoted in the previous version is accordingly withdrawn, and no magnitude-based figure of merit is claimed at the resonance. We treat the magnitude contrast at 16 MHz as a preliminary indicator only. The evidence that the two liquids present different loads to the transducer rests on the reactive component, which the Nyquist representation of Figure 6 displays directly and which is analysed in the following paragraphs. Read with that restriction, the observation is consistent with reports that APTES layers modify the interfacial response of impedimetric transducers [32,33,34]. The relative impedance variation decreased with increasing frequency, which is consistent with the frequency-dependent behavior reported for electrochemical biosensors [35,36].

There is a physical reason why the resonance minimum is the least informative point in the window, and it follows directly from the equivalent circuit of Figure 7. At series resonance the reactances of the electrode inductance L and the gap capacitance Cg cancel, so the impedance collapses onto the series resistance Rs of the electrode fingers, pads and leads. Rs is a property of the metal path and is not modified by the droplet. The two circuit elements that the sample does modify, Cg and Gd, act on the reactance rather than on Rs, and their effect on |Z| is therefore suppressed at precisely the frequency at which the reactance vanishes. The magnitude minimum is a transducer property, as argued above; it is not a sensing optimum, and the near-coincidence of the DI2ul and Nos2ul magnitudes there is expected rather than anomalous.

The reactive component behaves quite differently, and within the same frequency window it separates the two liquids unambiguously. On the ethanol-coated sensors the difference in −Im{Z} between the DI2ul and Nos2ul conditions reaches approximately 15.7 ± 1.8 MΩ near 6.8 MHz, a separation of roughly nine times the propagated chip-to-chip uncertainty, whereas at 16 MHz the difference in Re{Z} is 0.5 ± 1.2 MΩ and is indistinguishable from zero. The phase behaves in the same way: the DI2ul and Nos2ul phases differ by approximately 33 ± 7° at 4.5 MHz and by 23 ± 5° at 8 MHz, and the difference falls to 12 ± 7° at 16 MHz. The same pattern holds for the other two surfaces, with separations in −Im{Z} of approximately 26 ± 7 MΩ on the toluene-coated and 37 ± 15 MΩ on the uncoated sensors near 4.5 MHz. The discrimination between the two liquids therefore resides in the reactance and is resolved by the Nyquist representation, not by the magnitude at resonance; this is the basis on which the difference between the deionized-water and spore-containing conditions is asserted in this work.

The Nyquist plots presented in Figure 6 display the negative imaginary impedance component (−Im{Z}) as a function of the real impedance component (Re{Z}) across the measured frequency range for all sensor configurations. Following the established convention in EIS, the imaginary part of the impedance is plotted with a negative sign on the vertical axis, that is, as −Im{Z} [37,38]. This convention arises from the fact that purely capacitive elements yield a negative imaginary impedance (Im{Z} < 0) in the frequency domain, since the impedance of an ideal capacitor is given by ZCap = 1/(jωC) = −j/(ωC). Plotting −Im{Z} therefore places capacitive semicircular arcs in the upper half of the complex plane, facilitating the intuitive interpretation of RC-type equivalent circuit elements and making the characteristic semicircular features more readily identifiable [39]. All impedance values in Figure 6 are reported in MΩ, on the same scale as the magnitudes of Figure 5. Figure 5 and Figure 6 are two representations of the same spectra. Since |Z| = √(Re{Z}^2^ + Im{Z}^2^), neither component of Figure 6 can exceed the magnitude of the same condition at the same frequency. This relation was checked numerically against the underlying records at every acquired frequency and for every sensor–condition combination, and no violation was found. The apparent conflict between the axis limits of the two figures arises solely because they are plotted over different frequency ranges: Figure 5 is restricted to the resonance region, while the large real-axis excursions of Figure 6 occur below approximately 4 MHz, outside the lower limit of the range Figure 5 displays. For completeness we also set out the provenance of these spectra. Each trace in Figure 6 is the mean of three independently fabricated chips of the same coating type, each measured under all three sample conditions as paired observations, and the uncertainties quoted in the text are propagated from the scatter across those three chips. The underlying frequency-domain records, from which both Figure 5 and Figure 6 are drawn, are available from the authors on request, so that the internal consistency of the two representations can be verified independently. An additional repetition of the entire series on a newly fabricated batch of chips would test batch-to-batch reproducibility, which the present single-batch design cannot address; it could not be carried out within the resources available for this revision and is included in the further work set out in Section 3.

The behaviour is instead described by the lumped R–L–C network of four elements shown in Figure 7. A series inductance L represents the electrode fingers, the contact pads and the connecting leads; a series resistance Rs represents the ohmic loss of that same path; a capacitance Cg represents the inter-finger gap together with the substrate and fixture capacitance; and a conductance Gd represents the ionic conduction of the deposited droplet, which stands in parallel with Cg. Every feature of Figure 6 maps onto this topology. Below resonance the response is dominated by Cg, so that Im{Z} is negative and the locus lies in the upper half-plane. Near 16 MHz the reactances of L and Cg cancel and the locus crosses the real axis at a value set by Rs. Above resonance L dominates, the sign of the reactance reverses and the locus continues into the lower half-plane, closing the loop traced between approximately 10 and 30 MHz. Of the four elements, only Cg and Gd can be modified by the sample, and the reduction in impedance observed on adding the suspension is accordingly read as an increase in Gd. We do not report fitted element values. Four coupled parameters cannot be constrained with meaningful confidence intervals by three chips measured at a single spore concentration; and the fitted value of Gd would in any case not resolve the question that matters here, since whether its increase originates in the spores or in residual electrolyte is precisely what the present dataset cannot determine. Fitting is therefore deferred to the concentration series described in Section 3, in which each element can be tracked against a controlled variable.

A notable distinction between the DI2ul and Nos2ul responses is observed across all three coating conditions (Figure 6b–d). The DI2ul traces consistently describe the larger locus at the low-frequency end of the sweep, whereas the Nos2ul responses are confined to a markedly smaller one; the absolute limits are those plotted in Figure 6b–d, which share the kilo-ohm scale of Figure 5. This systematic reduction in impedance magnitude for Nos2ul relative to DI2ul, which is also apparent as a downward shift in the Bode |Z| curves of Figure 5, requires comment, since a population of micrometre-scale, poorly conducting bodies introduced into the inter-electrode gap would on its own be expected to raise rather than lower the impedance. The direction of the observed change indicates that the dominant effect is not the volume occupied by the spores but an increase in the ionic conductivity of the droplet. Deionized water has an extremely low conductivity, of the order of 1 µS/cm, so that even a small quantity of electrolyte carried over with the spore suspension residual ions from the isolation procedure, together with ionic material associated with the spore coat will dominate the bulk conductance of a 2 µL droplet. On this reading the measured signal reports the total ionic and dielectric load of the added suspension, of which the spores are one component among several. The present dataset does not contain the control that would separate these contributions, namely a spore-free supernatant obtained by centrifugation or filtration of the same suspension and measured under identical conditions. We identify this as the single most important experiment required before any spore-specific interpretation of the signal can be sustained. The same control should be accompanied by two further measurements that the present dataset also lacks: the conductivity of the spore suspension and of the deionized-water reference, recorded with a calibrated conductivity meter, and a preparation of spores washed repeatedly and resuspended in fresh deionized water, measured under identical conditions. Together these would establish how much of the observed contrast survives once the ionic background is equalized. The implications for the scope of the present claims are discussed in Section 3. The differential impedimetric response between DI2ul and Nos2ul is most pronounced for the ethanol-coated sensor (Figure 6d), where the geometric separation between the two Nyquist traces is visually most distinct. This observation is consistent with a chemical contribution from the ethanol-deposited film, but it does not establish one, since the same films also wet more extensively and therefore place a greater electrode length in contact with the sample for a fixed droplet volume.

Taken together, the Nyquist analysis provides clear insight into the distinct behavior induced by the APTES coatings and by the presence of *Nosema* (Figure 6). As shown in Figure 6a, uncoated and coated sensors without any applied solution did not exhibit appreciable differences in their impedance characteristics. However, once distilled water was applied (Figure 6b–d), the impedance characteristics began to differentiate according to coating type, and the most notable distinction was observed upon application of the *Nosema*-containing solution to the ethanol-coated sensor (Figure 6d). These results confirm that the sensor impedance is altered upon contact with a biological agent. Crucially, this change must be distinctive enough to differentiate the target analyte (*Nosema* spores) from the carrier liquid (distilled water). The ethanol-based APTES sensors displayed a larger impedance change in response to the spore-containing suspension than to distilled water alone. We note that this comparison establishes contrast between two liquids and not selectivity: no non-target particle, microorganism or spore-free filtrate was tested, and the term selective is therefore not applicable to the present data.

A question that follows directly from these comparisons is whether the APTES layer is necessary for the response at all. The present data answer it, and the answer is a qualified one that we state plainly. A separation between the deionized-water and the spore-containing conditions is present on every surface tested, including the uncoated control: the difference in −Im{Z} near 4.5 MHz is approximately 37 ± 15 MΩ on uncoated chips and 26 ± 7 MΩ on toluene-coated chips, against 15.7 ± 1.8 MΩ near 6.8 MHz on ethanol-coated chips. Functionalization is therefore not a precondition for the transducer to distinguish the two liquids, and we do not claim that it is. What changes with the coating is the reliability of the separation rather than its size. Expressed as a ratio to its own propagated chip-to-chip uncertainty, the separation is approximately 2.5 on uncoated sensors, 3.7 on toluene-coated sensors and 8.7 on ethanol-coated sensors. On this evidence the contribution of the ethanol-deposited film is to reduce device-to-device scatter, plausibly by producing a more uniform and more wettable surface across the interdigitated area, rather than to introduce a recognition mechanism directed at the analyte. This is a functional argument drawn from the impedance data themselves. It is not chemical evidence: it does not demonstrate that an aminopropylsiloxane layer is present, and it does not establish that such a layer is mechanistically required. Settling that question needs the spectroscopic characterization described in Section 2.2 together with a direct comparison of spore retention on the three surfaces after an identical wash, and both are deferred to the continuation of this work for the reasons given there.

### 2.4. Design and Implementation of a Portable Impedance Measurement System

In this study, a sensor capable of responding to *Nosema* spores was designed and experimentally evaluated. The performance of the sensor was validated using a benchtop impedance spectrometer connected to a computer. Although benchtop impedance spectrometers provide high measurement accuracy, they are costly to procure and to operate, and they typically require a well-equipped laboratory environment.

In order to overcome these limitations, the development of a portable and cost-effective system capable of detecting *Nosema* spores without the need for laboratory-based measurements or substantial investment in spectrometer infrastructure was considered highly advantageous. Consequently, a system based on a single-board computer and a microcontroller was designed and fabricated. A schematic representation of the overall system architecture is presented in Figure 8.

The portable impedance measurement system is based on an ESP32 microcontroller and an AD9833 direct digital synthesis (DDS) signal generator. The ESP32 controls the AD9833 via the SPI communication protocol and applies sinusoidal excitation signals at different frequencies to the sensor. Since the output of the AD9833 is unipolar, the generated signal passes through an amplification and offset-adjustment stage in order to obtain a bipolar and symmetric waveform. Variations in the sensor impedance cause changes in the current flowing through the sensor, and a transimpedance amplifier converts this current into a voltage signal.

The resulting signal undergoes signal conditioning, comprising amplification, half-wave rectification using a diode-based rectifier, and low-pass filtering through an RC network. This process converts the sinusoidal signal into a DC voltage proportional to its amplitude. A voltage divider scales this voltage to match the input range of the ESP32 analog-to-digital converter (ADC), where the signal is digitized. The ESP32 acquires the measurement data at a defined sampling interval and transmits the data to a single-board computer via Wi-Fi using the UDP protocol. The device incorporates an SSD1306 OLED display to present measurement values and system parameters, and a rotary encoder together with push buttons enables user interaction and parameter configuration directly on the device (Figure A1).

The device was connected to the previously fabricated sensor chip as shown in Figure A2. A sinusoidal excitation signal with a frequency of 1 MHz and an amplitude of 1 V was applied to the sensor. The impedance minimum located near 16 MHz in the benchtop measurements is a property of the interdigitated electrode structure itself and served in Section 2.3 as a common reference point for comparing the three coating chemistries; it is not, however, the frequency at which the contrast between the two liquid samples is largest. The Nyquist representations of Figure 6b–d show that the DI2ul and Nos2ul loci diverge most strongly in the low-frequency region, where the DI2ul locus is substantially the larger of the two. The 1 MHz operating point of the portable unit, which is set by its DDS and signal-conditioning stages, therefore falls within the frequency window that the benchtop Nyquist analysis identifies as the most informative for distinguishing the analyte from the carrier liquid. After the system had reached a stable condition, 2 µL of pure water was deposited onto the sensor surface in order to obtain a reference measurement. Since pure water does not cause a large impedance variation at this frequency, the measured output voltage was approximately 120 mV.

After the preliminary measurement, the sensor surface was cleaned and 2 µL of a solution containing approximately 3000 *Nosema* spores was applied to the sensor. Following this application, the measured voltage increased to approximately 160 mV. The rise in output voltage corresponds to a fall in sensor impedance and hence to a larger current through the sensor. We do not attribute that fall specifically to a spore–surface interaction. As set out in Section 2.3, the suspension differs from the deionized-water reference in its total ionic content as well as in its particulate content, and the portable experiment includes no spore-free control capable of separating the two contributions.

As shown in Figure 9, the output voltage of the portable device rises from a baseline of approximately 120 mV to approximately 160 mV following application of the *Nosema*-containing solution, a relative change of approximately 33%. We deliberately do not convert this figure into a percentage change in impedance. Although the front end is a transimpedance stage, for which V*_out_* is inversely proportional to Z under ideal conditions, the value displayed by the instrument is produced only after further amplification, half-wave rectification by a diode, RC low-pass filtering and resistive division. Diode rectification in particular is neither offset-free nor strictly linear at these amplitudes, so the mapping from the sinusoidal amplitude at the sensor to the digitized DC level is not guaranteed to be proportional or to pass through the origin. Converting a 33% voltage change into an impedance change would require the complete chain to be calibrated against a set of known impedances, which was not done. The portable result is therefore reported as an uncalibrated voltage change of a definite sign—the output rises, indicating that the sensor impedance falls—and the earlier statement that it corresponds to a 25% impedance reduction has been withdrawn. For the same reason it is not compared numerically with the magnitude contrast obtained on the benchtop instrument: the two measurements differ in excitation frequency (1 MHz against 16 MHz), in excitation amplitude (1 V against 100 mV) and in signal chain. What the two platforms establish in common is the direction of the effect, namely that introducing the spore suspension lowers the sensor impedance. We describe this as agreement in direction between two instruments rather than as cross-validation, since the latter would require repeated paired measurements on the same samples at a common frequency, which were not performed.

The record in Figure 9 spans approximately 20 min, which is the interval over which the output was monitored in order to establish that the post-application level was stable; it is not a response time. In the previous version of this manuscript the transient was described by a fourth-order polynomial fit and a response time of 60 s was quoted. Both have been withdrawn, and the claim of a 60 s response has been removed from the abstract, the discussion and the conclusions as well as from this section. A polynomial of that order carries no mechanistic meaning, and its high coefficient of determination reflects only the flexibility of the function. More importantly, the record contains fluctuations comparable in amplitude to the step itself, so that neither an initial baseline nor a final plateau can be defined with sufficient confidence to place a t_90_, the time to reach 90% of the total excursion. Because the experiment consists of one sequential measurement on a single sensor with a single sample, no replicate exists from which an uncertainty on such a value could be obtained, and quoting a single number would misrepresent the evidence. We therefore report no response time. Establishing one will require repeated loadings across several devices, with the instant of sample application logged by the acquisition software rather than inferred from the trace, and with the endpoint defined in advance; this is set out among the further work in Section 3. The transient of Figure 9 should accordingly be read as illustrating the shape and the sign of the response, and nothing more. Device-to-device variation, drift over successive loadings and the influence of the intervening cleaning step were likewise not assessed.

## 3. Discussion

Biosensors are emerging as powerful tools across diverse fields such as biomedical diagnostics, environmental monitoring, pharmaceutical delivery, food safety, and disease progression tracking [2]. Among biosensor technologies, impedance-based electrochemical methods offer advantages including high sensitivity, simplicity, cost-effectiveness, rapid response, and field applicability [21,22,40,41,42]. Their portability makes them attractive candidates for on-site analysis, particularly in agricultural and resource-limited settings. In the present study an impedimetric transducer was fabricated and characterized, and its response to aqueous *Nosema* spore suspensions was evaluated. Detection of spores in honeybee feces is the application that motivates the work, not something these data demonstrate: no fecal sample, authentic or simulated, was measured, and the term biosensor is reserved here for platforms carrying a molecular recognition element, which APTES is not. *Apis mellifera* is a keystone pollinator that is vital for ecological balance and crop productivity. Diseases such as those caused by *Nosema* spp. threaten bee health directly and may also pose an indirect risk to humans, since the antimicrobial agents used to control *Nosema* infections can leave residues that contaminate hive products [28].

While molecular methods offer high specificity, light microscopy is less reliable for species differentiation and may fail to resolve early developmental stages such as sporonts, sporoblasts and immature spores, or to detect low infection levels. This is particularly problematic when antibiotic-resistant *Nosema* strains are considered, since incorrect or delayed identification can lead to ineffective treatment and consequent economic loss [18,24,30]. In addition, relying solely on spore counts from pooled samples may not accurately reflect the true infection status of a colony. Recent advances in mobile and portable diagnostic devices have explored microfluidics and smartphone-based fluorescence imaging, but many of these approaches still rely on invasive sampling or on complex reagents [18,25,30,43,44]. The platform proposed here is directed toward the same goal, namely real-time and non-destructive detection of mature spores in material shed by infected bees. That application is the motivation for the work rather than a result of it: no fecal sample, whether authentic or simulated, was measured in this study.

The performance of the sensor relies on laser-fabricated interdigitated electrodes functionalized with APTES. The results indicate that ethanol-based APTES coatings provide improved performance relative to toluene-based or uncoated sensors. The enhanced hydrophilicity obtained with the ethanol solvent is the proximate cause, but it operates through two mechanisms that the present design cannot separate: more uniform droplet dispersion, which enlarges the wetted electrode area, and any improved interaction between the spores and the functionalized surface. Separating them requires the droplet footprint to be fixed rather than allowed to follow the wettability of the coating, for example by confining the sample within a hydrophobic ring of defined diameter so that the contacted electrode length is identical on all three surfaces, and by comparing spore retention on uncoated, ethanol-APTES and toluene-APTES surfaces after an identical wash, counted microscopically. Both are straightforward and are planned; neither was performed here. The electrochemical stability and effectiveness of ethanol-based APTES coatings have also been demonstrated in other studies involving detection in complex matrices such as seawater, and in applications requiring long-term interfacial stability [26,45]. These findings indicate that laser-fabricated, APTES-functionalized interdigitated electrodes constitute a workable transducer platform, and that the impedance contrast they produce between a spore suspension and a water control is large enough to be resolved without laboratory instrumentation. They do not establish detection in the analytical sense, which would require a demonstrated relationship between signal and spore concentration, a defined limit of detection, and evidence that the signal is attributable to the spores rather than to the ionic composition of the suspension.

In addition to the impedance spectra, the time-dependent voltage response obtained with the portable unit illustrates what a self-contained readout of this platform can record. The unit is intended for eventual field deployment and requires no laboratory infrastructure to operate. It registers a change of definite sign following exposure to the *Nosema*-containing sample, and the output then settles. No response time is quoted. The statement in the previous version of this manuscript that a stable signal change occurred within 60 s has been withdrawn, for the reasons given in Section 2.4: the record is a single transient on one device, its fluctuations are comparable in amplitude to the step, the instant of sample application was not independently logged, and no replicate exists from which an uncertainty could be derived.

Following the initial shift the voltage settles to a steady level. We do not interpret that plateau as the completion of surface binding or as the establishment of a binding equilibrium at the sensor–liquid interface. Such an interpretation would require evidence that the signal originates from spores attaching to the functionalized surface, which the present experiments do not provide; a plateau is equally consistent with the droplet reaching thermal and ionic equilibrium across the electrode gap. What the portable measurement demonstrates is that the impedance contrast between the two liquids is large enough to be resolved by inexpensive electronics, which is a statement about the transducer and its readout rather than about the mechanism. Conventional microscopic and molecular techniques remain the reference methods; they are time-consuming, invasive and dependent on laboratory infrastructure, and the interest of the present platform lies in the possibility of eventually complementing them with a non-destructive on-site measurement, not in having done so.

A point that merits explicit comment concerns the frequency at which the two measurement platforms operate. The impedance minimum near 16 MHz reported in Section 2.2 originates from the parasitic capacitance and inductance of the interdigitated electrode geometry and therefore reflects a property of the transducer rather than of the analyte. It provides a convenient common reference point at which the three coating chemistries can be compared on an equal footing, but it does not follow that this frequency is also the one at which the contrast between the two liquid samples is greatest. The Nyquist representations of Figure 6b–d indicate the opposite: the DI2ul and Nos2ul loci diverge most strongly at the low-frequency end of the measured window, where the impedance is governed by the electrical double layer and by the ionic conductivity of the deposited droplet, that is, by precisely those properties that the presence of spores modifies. As the excitation approaches the structural resonance, the response becomes progressively dominated by the reactive elements of the electrode geometry, which are insensitive to the composition of the liquid. The 1 MHz operating point of the portable unit is therefore not merely a constraint imposed by its signal-generation hardware; it lies within the frequency window that the benchtop Nyquist analysis identifies as the most informative for discriminating the analyte from the carrier liquid.

It should nevertheless be emphasized that the uncalibrated voltage change recorded by the portable device at 1 MHz and the impedance magnitude contrast obtained from the benchtop spectrometer at the 16 MHz reference point are not interchangeable quantities, since they are acquired at different excitation frequencies, at different excitation amplitudes (1 V against 100 mV) and through entirely different measurement chains, only the first stage of which is a transimpedance converter. The two results should accordingly be read as mutually corroborating in direction rather than in magnitude. Their agreement on the sign of the effect—a reduction in sensor impedance upon exposure to *Nosema* spores—obtained on two independent instruments, is what supports the robustness of the sensing principle and, by extension, the feasibility of transferring the measurement from a laboratory spectrometer to a field-deployable unit.

The limitations of this study are extensive, and we set them out in full because they define the scope within which the results should be read. Stated at its most conservative, what the benchtop data establish is that a *Nosema*-containing suspension presents a different ionic and dielectric load to the transducer than ultrapure water does. We accept that description of the finding, and the claims made throughout this revision are framed so as not to exceed it. First, and most fundamentally, the origin of the impedance change has not been established. The only liquid control was deionized water; no spore-free filtrate or supernatant from the same suspension was measured, no washed-spore preparation was tested, and the conductivities of the suspension and of the control were not recorded. The observed decrease in impedance is at least as consistent with an increase in the ionic conductivity of the droplet as with an interaction between the spores and the functionalized surface, and the present data cannot distinguish between the two. Second, all measurements were performed at a single spore concentration of 1500 spores/µL, so that no calibration series, working range, limit of detection or limit of quantification is available and the analytical sensitivity of the platform can be neither stated nor compared with haemocytometer counting or quantitative PCR. Third, selectivity was not assessed: no non-target particle, microorganism, pollen grain or component of honeybee gut content was measured, and APTES supplies a general amine-terminated surface rather than a recognition element directed at *Nosema*, so nothing in these data excludes comparable responses from other particulate or ionic material. Fourth, no measurement was performed in a fecal matrix, authentic or simulated; honeybee feces have a far higher and more variable ionic strength than deionized water, and a conductivity-driven signal of the kind observed here would be expected to be substantially attenuated, if not abolished, in such a matrix. Fifth, the improved contrast obtained with ethanol-deposited APTES remains confounded with droplet spreading, since the more hydrophilic surface increases the wetted electrode area for a fixed droplet volume, and the chemical and geometric contributions have not been separated.

Sixth, the replication structure limits what can be inferred about reproducibility. The three repeats of each configuration are device-level replicates drawn from a single fabrication batch; no batch-to-batch comparison was made. The magnitude contrast at the 16 MHz reference point, approximately 10%, is smaller than its own propagated uncertainty of approximately 13% and is therefore not resolved; this is stated explicitly in Section 2.3 and no analytical claim is made from it. The separation in the reactive component near 6.8 MHz exceeds its uncertainty by about a factor of nine and is the quantity on which the comparison between the two liquids rests, but it is subject to the same unresolved question of origin set out above, since nothing in these data establishes that the reactive difference is produced by the spores rather than by residual electrolyte. Seventh, the portable-device experiment rests on a single sequential measurement on one sensor, so that drift, carry-over across the intervening cleaning step and device-to-device variability were not evaluated, and no response time can be extracted from it. Eighth, every measurement was made at 21 °C inside a Faraday cage, whereas apiary conditions involve variable temperature and humidity, ambient electromagnetic noise and heterogeneous samples, none of which was simulated; the portable electronics represent a step toward field use and do not constitute a field-validated instrument. Ninth, gold was deposited directly onto glass without a titanium or chromium adhesion layer, which may compromise electrode integrity over repeated measurement cycles, and the cleaning procedure applied between successive loadings was itself not validated for carry-over or for film degradation. Tenth, the presence, chemical identity and surface coverage of the APTES film were not confirmed by a chemically specific technique. Contact-angle goniometry and scanning electron microscopy establish that the two solvent treatments leave surfaces of different wettability and comparable morphology, but neither demonstrates that an aminopropylsiloxane layer is present, and X-ray photoelectron or infrared spectroscopy would be required for that purpose. The omission is one of access rather than of intent: neither technique was available within the funding and instrumentation of the present project. The same constraint applies to the independent repetition of the impedance series on a second fabrication batch. Both are the first items of the planned continuation, and until they are completed the role assigned to the APTES film here remains a functional inference from the impedance data rather than a chemically demonstrated one. Eleventh, the assignment of the impedance minimum near 16 MHz to a structural resonance rests on the internal consistency arguments given in Section 2.3 rather than on a dedicated experiment; varying the lead length and measuring the empty fixture would test it directly, and neither was done. Addressing the first four of these limitations a spore-free supernatant control measured alongside solution conductivities, a concentration series under a common ionic background, a non-target panel and measurement in a fecal matrix constitutes the programme of work required to convert the present transducer demonstration into an analytical method, and is the focus of ongoing experiments.

## 4. Conclusions

Three results are reported. A nanosecond laser-ablation route patterns interdigitated gold electrodes on ordinary microscope slides without photolithography or cleanroom access, and the electrodes so produced are electrically functional across 0.01–50 MHz. APTES films deposited from ethanol yield a larger impedance contrast between an aqueous spore suspension and a deionized-water control than films deposited from toluene or than bare electrodes, although this comparison remains confounded with the greater spreading of the droplet on the more wettable surface. That contrast is large enough to be resolved by a self-contained microcontroller readout costing a small fraction of a benchtop spectrometer, which registers a change of the same sign. Each of these is a statement about the transducer and its instrumentation. None is a diagnostic result. All measurements were made under controlled laboratory conditions at 21 °C inside a Faraday cage, at one spore concentration, in deionized water, without a spore-free control, without a selectivity panel and without any fecal matrix; the platform has therefore not been shown to operate outside a laboratory, and no response time is claimed for it. Establishing that the signal originates from the spores, constructing a calibration function with a defined limit of detection, and demonstrating that the response survives a fecal matrix are each necessary conditions for the platform to become a diagnostic tool, and none has yet been met. The work is reported at this stage because the fabrication route and the portable readout are of independent methodological interest, and because the frequency-domain analysis presented here indicates where in the spectrum such an assay should subsequently be operated: at the low-frequency end of the window, where the response is governed by the conductivity and permittivity of the sample, rather than at the structural resonance, where it is governed by the geometry of the electrode. The simplicity of the fabrication process also lends itself to integration with microfluidic sample handling and to scaled production.

## Figures and Tables

**Figure 1:**
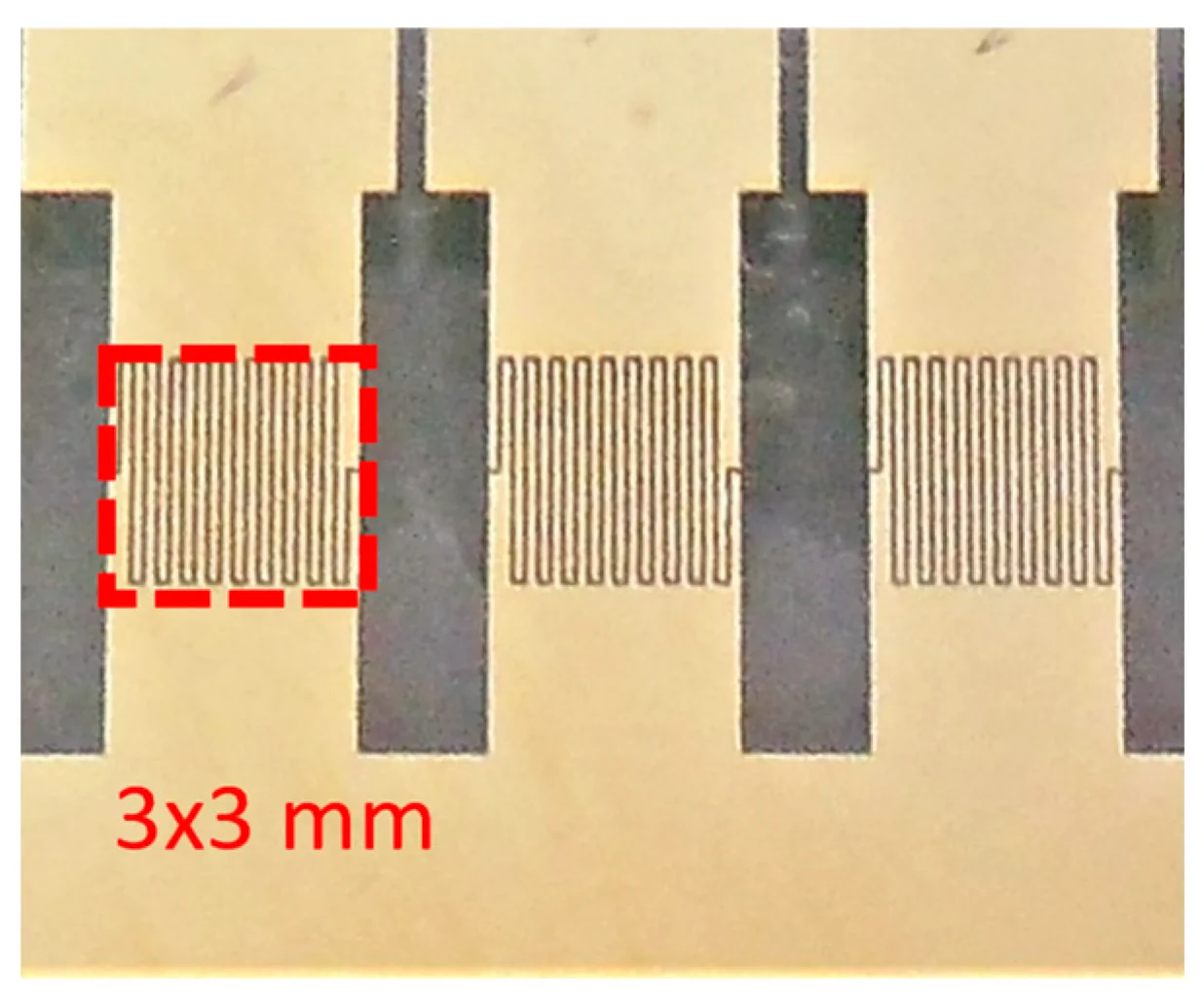
Three adjacent 3 × 3 mm interdigitated electrode (IDE) sensor structures. The box enclosed with a dashed red line indicates one active area.

**Figure 2:**
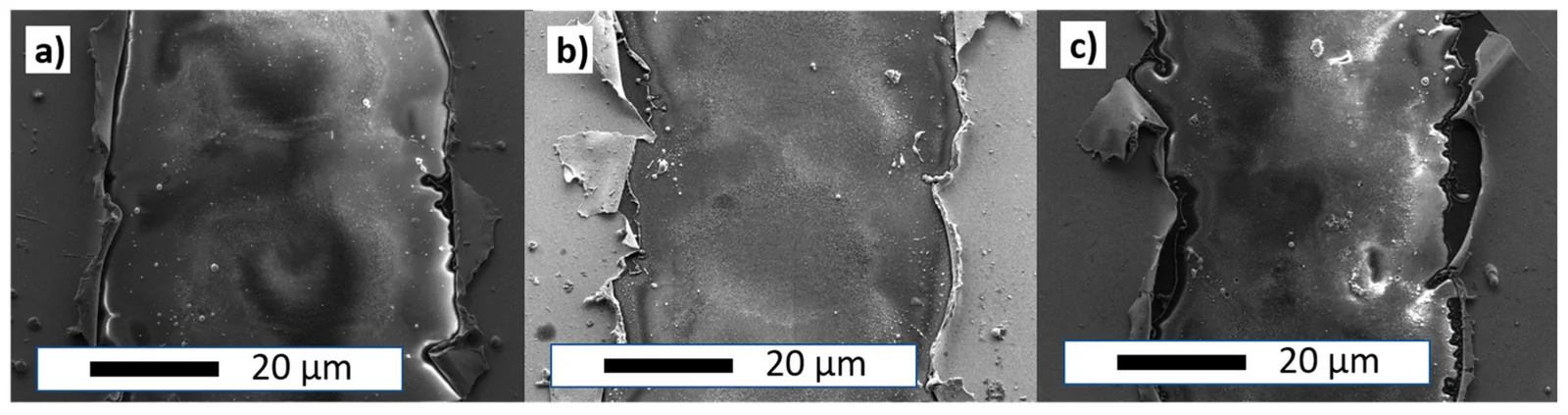
SEM images of sensor surfaces coated with different solutions. The central regions are glass and the lateral regions are gold-coated: (**a**) uncoated glass, (**b**) glass coated with ethanol-based APTES, and (**c**) glass coated with toluene-based APTES. The left and right portions of each image show the 100 nm gold-coated areas, while the central gaps are the laser-ablated regions exposing the glass substrate. Irregularities are visible at the gold–glass boundaries, most likely arising from thermal effects during laser ablation and from imperfections associated with depositing gold directly onto glass without an adhesion layer.

**Figure 3:**
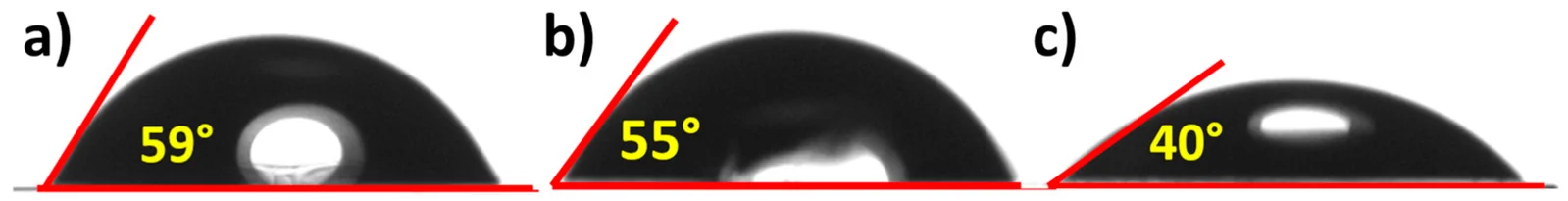
Static water contact angle (WCA) measurements of the IDE sensor surfaces: (**a**) uncoated reference (59°), (**b**) toluene-coated (55°), and (**c**) ethanol-coated (40°). Red lines denote the contact angle tangents.

**Figure 4:**
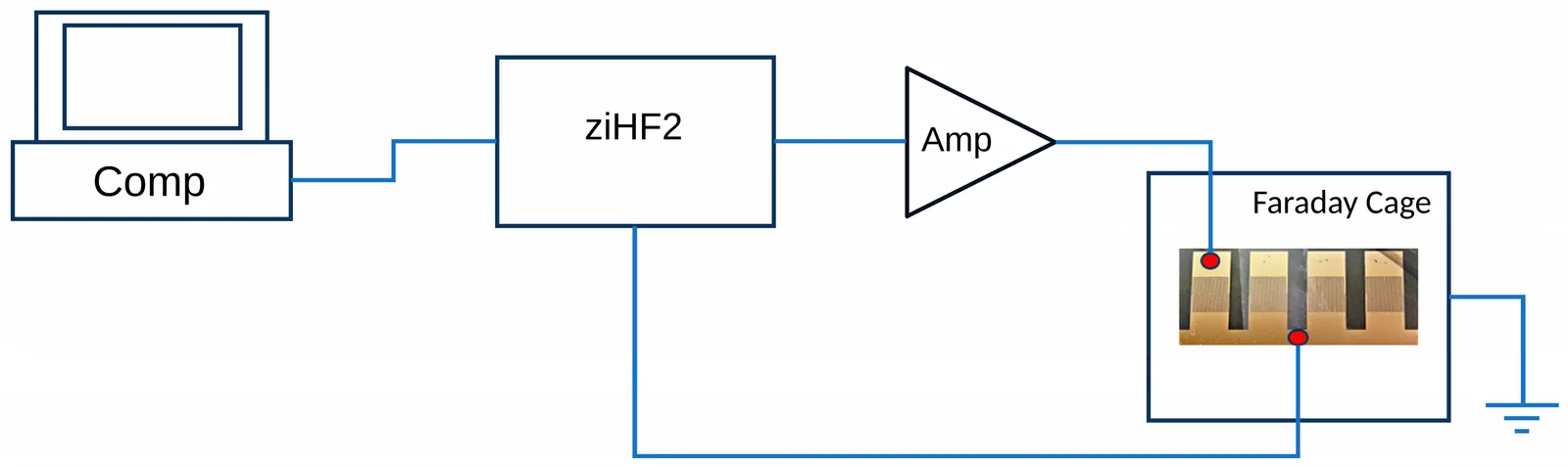
Experimental setup and workflow outline for the impedance measurements of the sensor arrays reported in this work.

**Figure 5:**
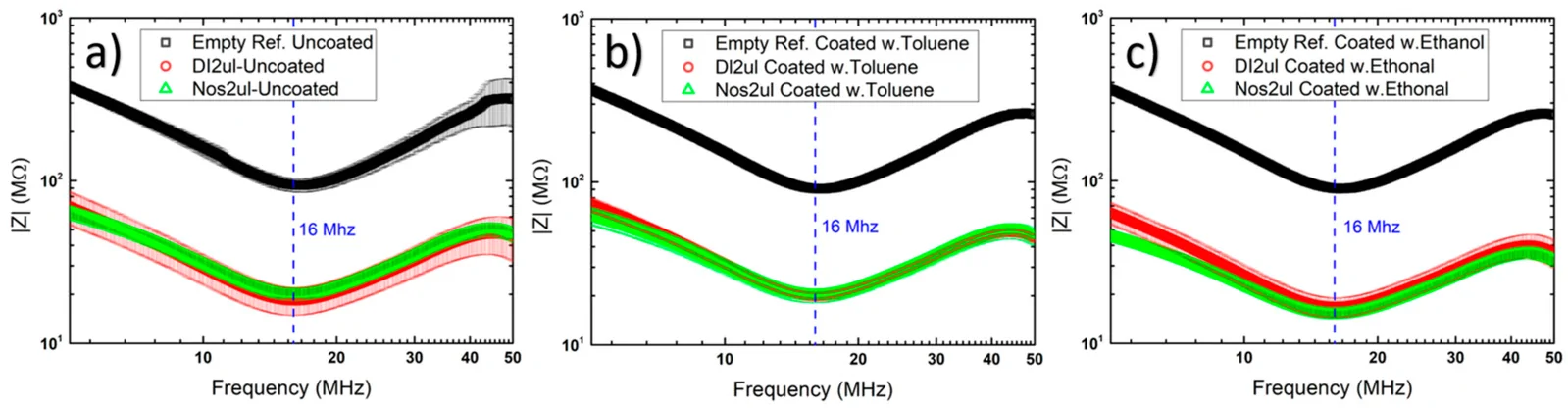
Bode plots of impedance magnitude versus frequency for the different sensor types: (**a**) uncoated, (**b**) APTES-coated with toluene, and (**c**) APTES-coated with ethanol. Legend: black square = Reference, red circle = DI2ul, green triangle = Nos2ul. The vertical axis is in MΩ. The dashed vertical line indicates the 16 MHz resonance frequency. The frequency axis is restricted to the region surrounding the resonance, which is the part of the window in which the lumped-element description of Section 2.3 applies and in which the three coatings are compared; Figure 6 displays the full measured window, so the two figures are drawn over deliberately different frequency ranges and their axis limits are not directly comparable. Each point is the mean of three sensor chips of the same coating type (*n* = 3) fabricated within a single batch and measured under all three sample conditions as paired observations; error bars denote the standard deviation across those three chips.

**Figure 6:**
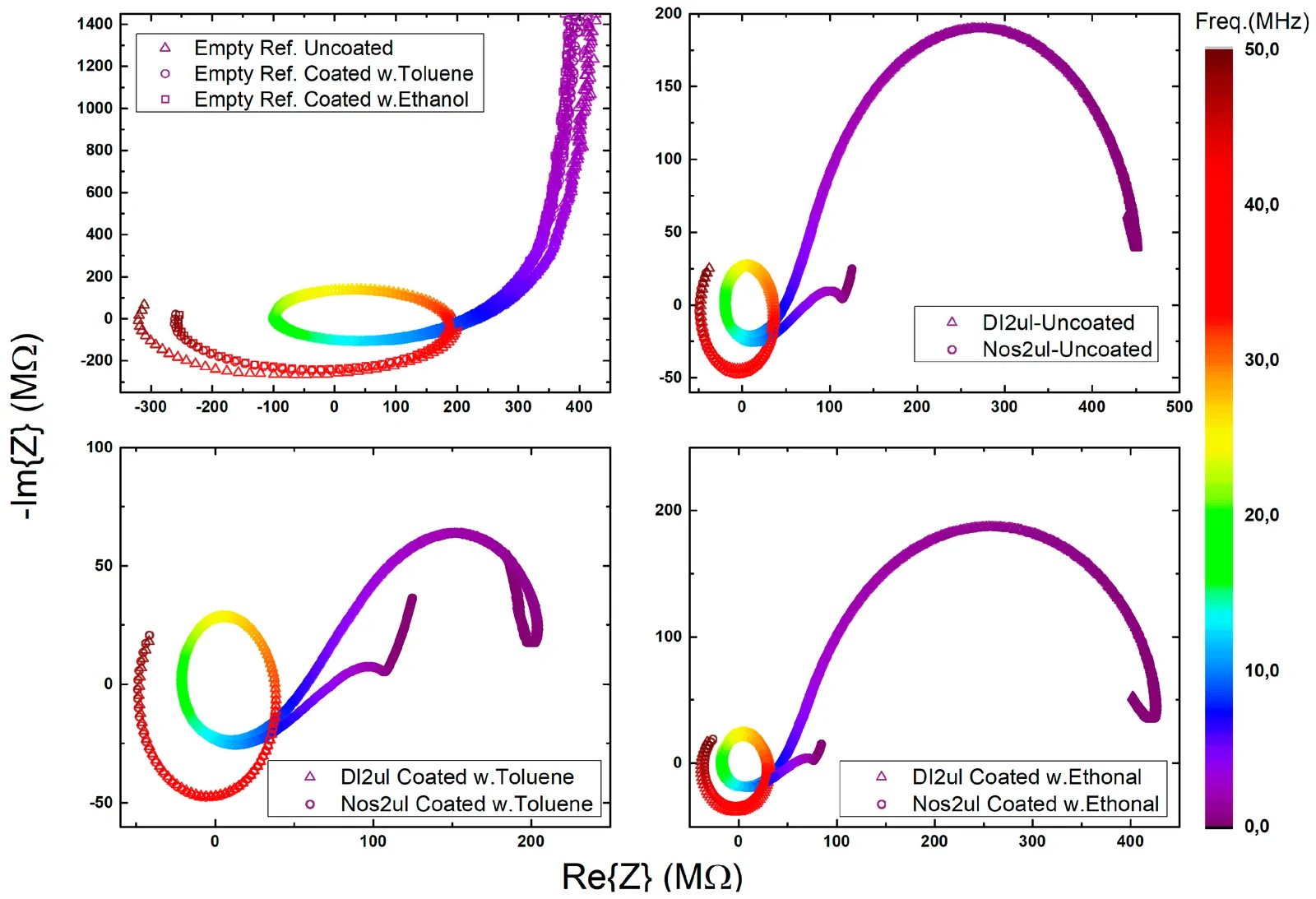
Nyquist plots (−Im{Z} versus Re{Z}) of the IDE sensors under different measurement conditions. (**a**) Empty reference measurements for the uncoated, toluene-coated, and ethanol-coated sensors. (**b**–**d**) Impedance responses upon loading with 2 µL distilled water (DI2ul) and 2 µL *Nosema*-containing solution (~3000 spores, Nos2ul) for (**b**) uncoated, (**c**) toluene-coated, and (**d**) ethanol-coated sensors. The frequency-encoded color scale (0.01–50 MHz) is shared across panels (**b**–**d**). All impedance values are given in MΩ. Each trace is the mean of three sensor chips of the same coating type (*n* = 3), measured as paired observations on the same devices; error bars are omitted from this representation for clarity, and the chip-to-chip standard deviations for the corresponding conditions are shown in Figure 5. The vertical axis of every panel is −Im{Z}, and the axis labels have been corrected accordingly in this revision. Panel (**a**) is scaled to resolve the structure near the resonance; the unloaded reference loci continue beyond the plotted area, reaching −Im{Z} of the order of 2 × 10^4^ MΩ and Re{Z} of approximately 1.6 × 10^3^ MΩ at the lowest measured frequencies. Figure 6 covers the full measured window of 0.01–50 MHz, whereas Figure 5 is restricted to the resonance region; the axis limits of the two figures are therefore not directly comparable, and the larger excursions visible here occur at frequencies below the range displayed in Figure 5.

**Figure 7:**
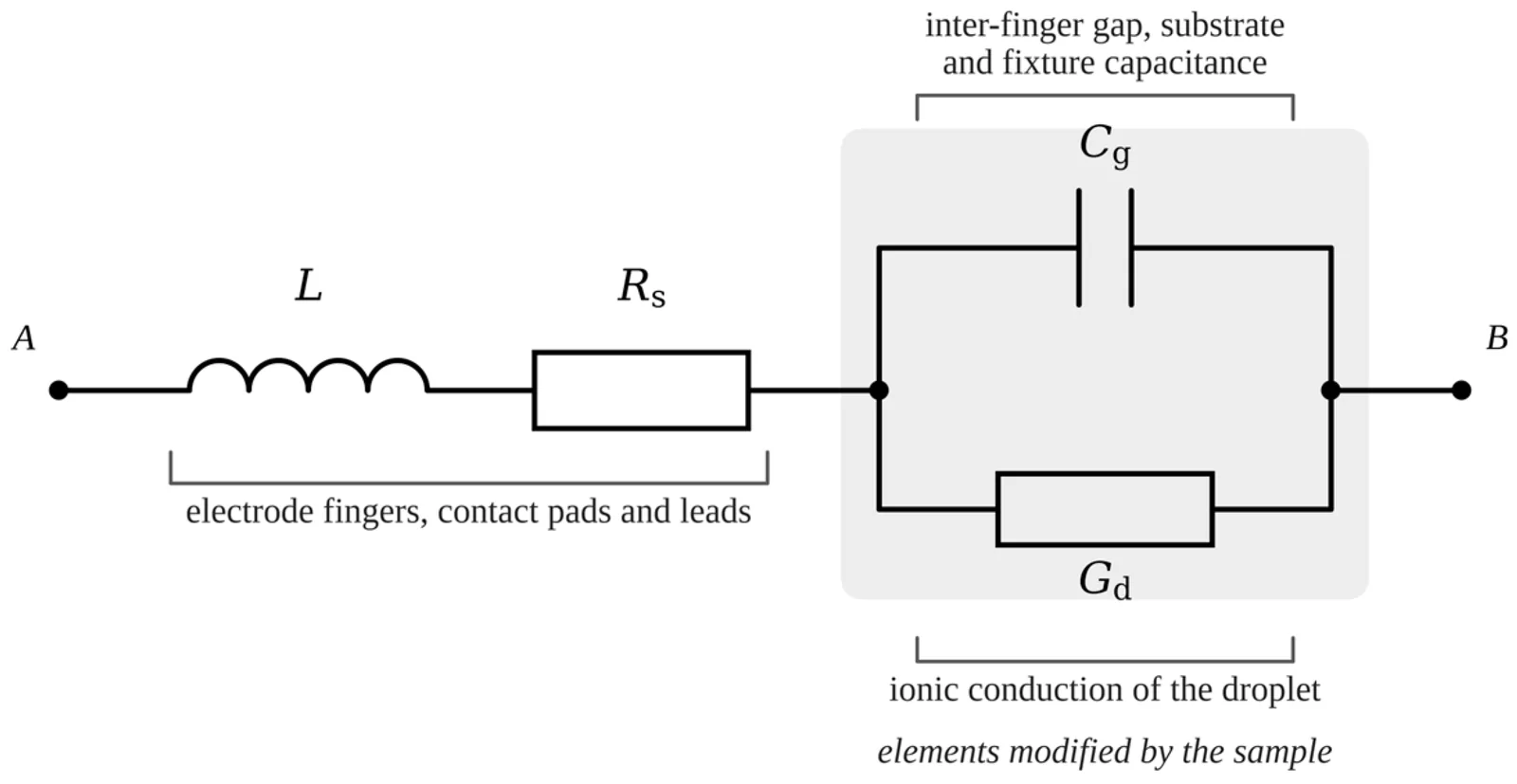
Lumped R–L–C equivalent circuit of the interdigitated transducer under liquid loading, between the two comb terminals A and B. The series inductance L and resistance Rs represent the electrode fingers, the contact pads and the connecting leads; the capacitance Cg represents the inter-finger gap together with the substrate and fixture capacitance; and the conductance Gd represents the ionic conduction of the deposited droplet, which stands in parallel with Cg. Only the two shaded elements can be altered by the sample. Series resonance occurs where the reactances of L and Cg cancel, at which point the impedance locus of Figure 6 crosses the real axis at a value set by Rs; below that frequency the response is capacitive and the locus lies in the upper half-plane, and above it, the response is inductive and the locus passes into the lower half-plane.

**Figure 8:**
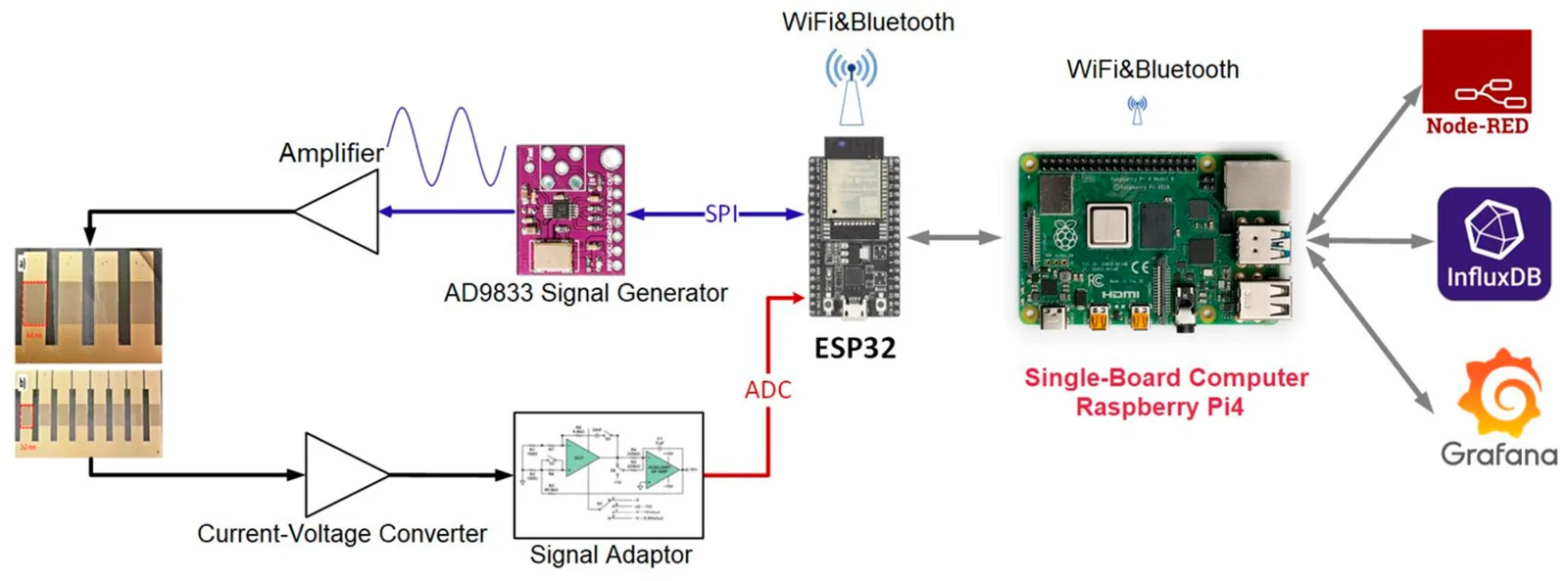
Architecture of the portable impedance measurement system.

**Figure 9:**
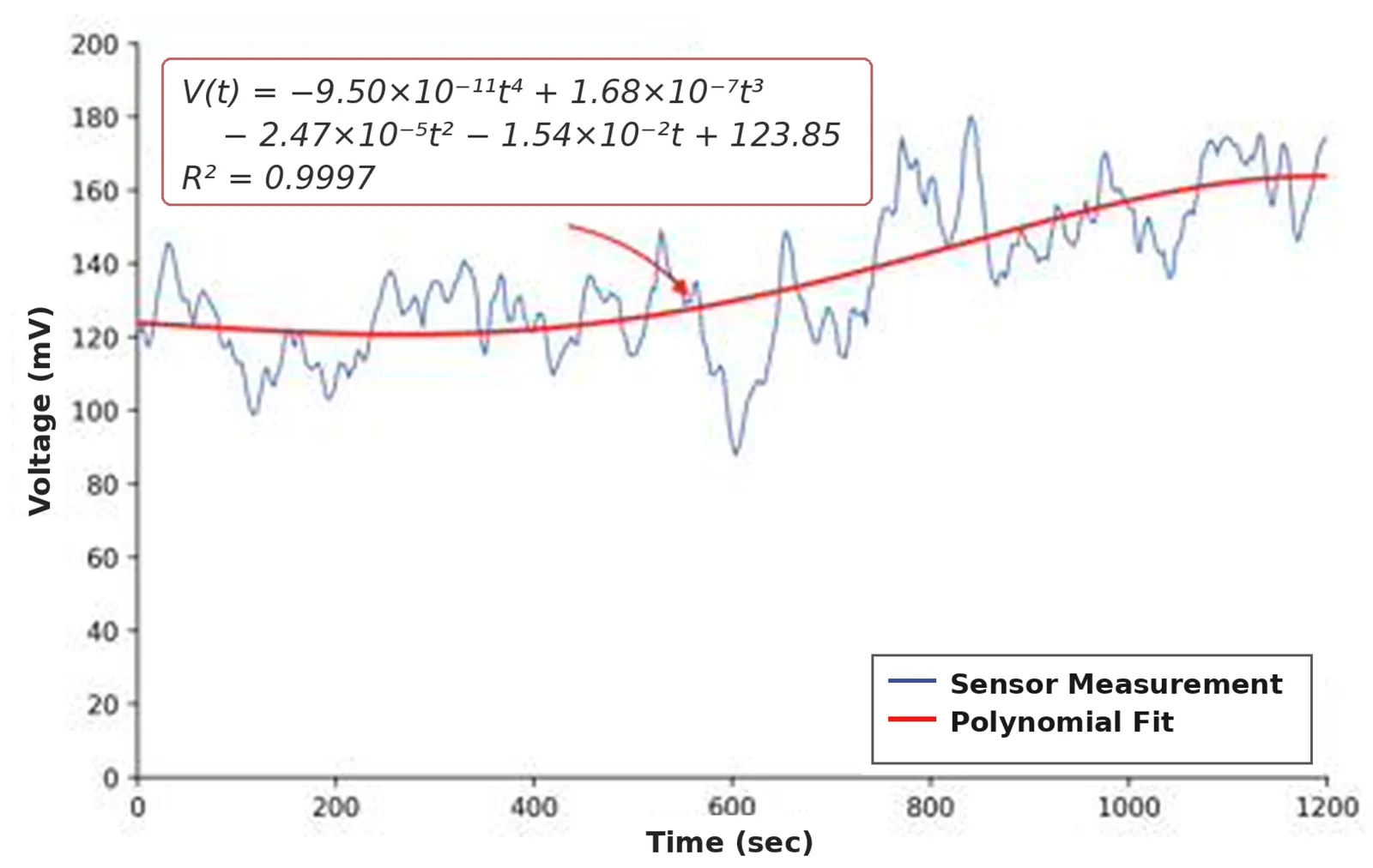
Output voltage of the portable device recorded continuously during a single sequential experiment on one ethanol-coated sensor. The trace is shown exactly as acquired; the fourth-order polynomial fit and its inset that appeared in the previous version have been removed. The arrow marks the instant at which the 2 µL spore suspension was applied, and the preceding portion of the record is the deionized-water baseline. No response time is derived from this record; see the accompanying text.

**Table 1:** Primers used for multiplex identification of *N. ceranae* and *N. apis*.

Primer	Sequence (5′→3′)	Product (bp)	Specificity
218MITOC-FOR	CGGCGACGATGTGATATGAAAATATTAA	218	*N. ceranae*
218MITOC-REV	CCCGGTCATTCTCAAACAAAAAACCG	218	*N. ceranae*
321APIS-FOR	GGGGGCATGTCTTTGACGTACTATGTA	321	*N. apis*
321APIS-REV	GGGGGGCGTTTAAAATGTGAAACAACTATG	321	*N. apis*

Primer sequences from Martín-Hernández et al. [11].

**Figure A1:**
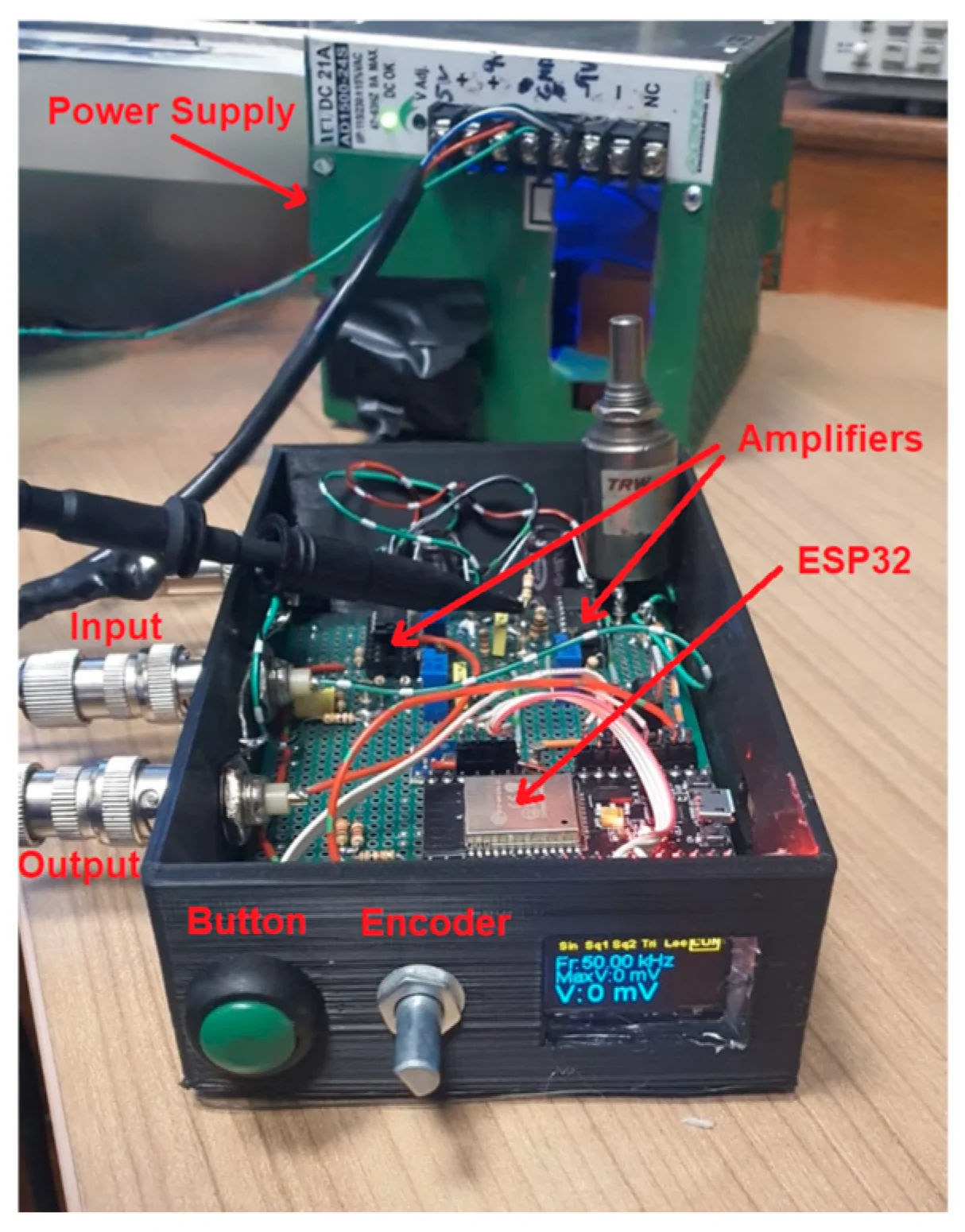
Photograph of the assembled portable impedance measurement device, showing the OLED display, the rotary encoder and the push buttons used for parameter entry.

**Figure A2:**
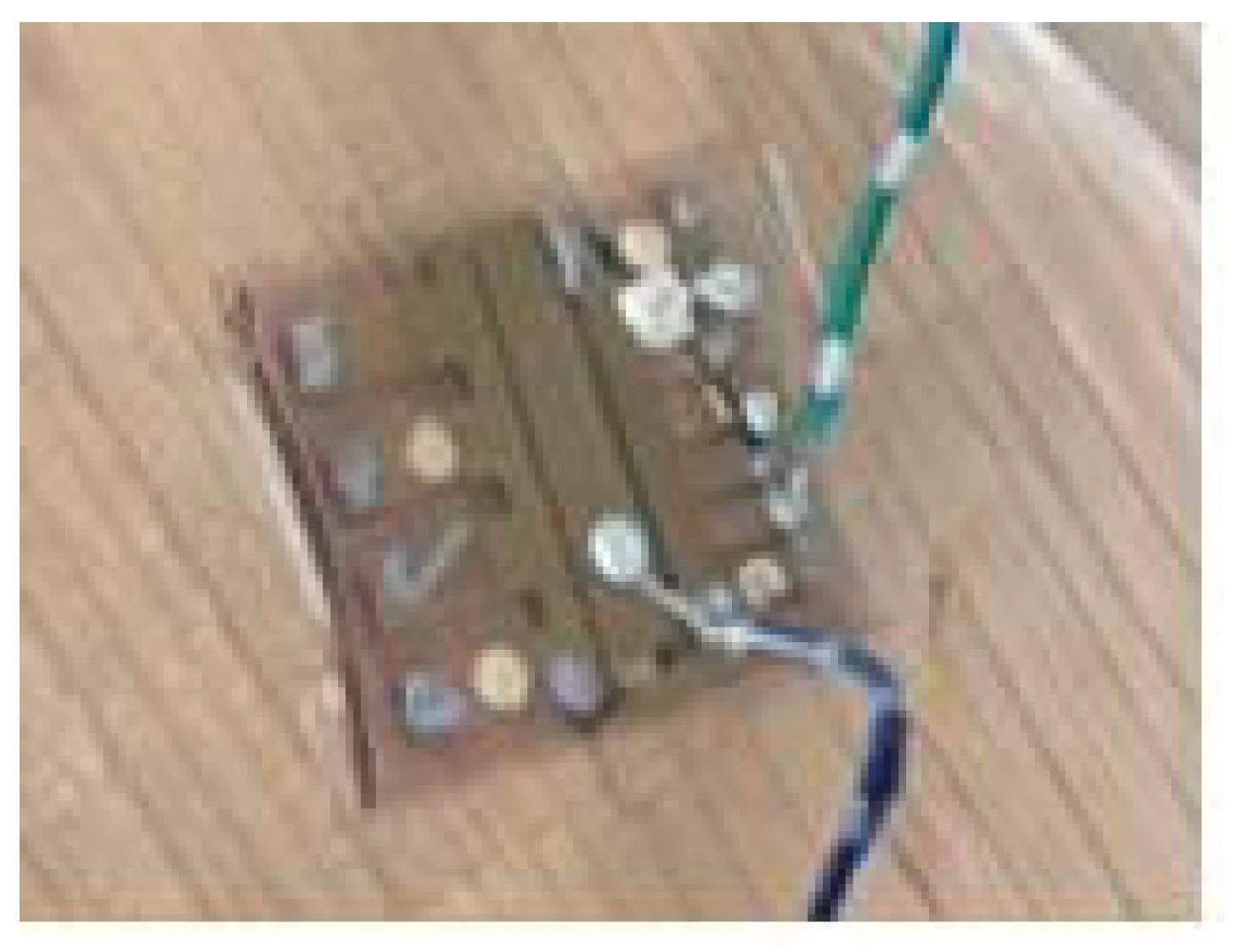
Photograph of a fabricated sensor chip carrying three adjacent interdigitated sensing areas.

## Data Availability

Data are available on request from the authors.
